# Comment on ‘Effects of Sarcopenia and Frailty on Postoperative Recovery in Elderly Patients: A Prospective Cohort Study’ by Guo et al.

**DOI:** 10.1002/jcsm.13625

**Published:** 2024-10-21

**Authors:** Hongrui Chen, Zening Huang, Qinqi Yu, Bin Sun, Chen Hua, Xiaoxi Lin

**Affiliations:** ^1^ Department of Plastic & Reconstructive Surgery Shanghai Ninth People's Hospital, Shanghai Ninth People's Hospital, Affiliated to Shanghai Jiao Tong University School of Medicine Shanghai China; ^2^ Department of Gastric Surgery Fujian Medical University Union Hospital Fuzhou China; ^3^ School of Stomatology Fujian Medical University Fuzhou China

Dear Editor,

We read with great interest the article by Guo et al [1]. This paper reported the results of a prospective cohort study exploring the impact of sarcopenia and frailty on postoperative recovery in elderly patients. The study found that elderly patients with sarcopenia and frailty experienced poorer recovery post‐surgery, characterized by a higher incidence of complications within 90 days, prolonged hospital stays and decreased postoperative self‐care ability.

Sarcopenia is identified as an independent risk factor for postoperative complications. In a comprehensive review article, the pathophysiology and clinical significance of sarcopenia were thoroughly outlined [2]. The decreased release of amino acids in the muscles of patients with sarcopenia may lead to a shortage of raw materials for acute‐phase protein synthesis, thereby affecting the activity of immune cells. Furthermore, the ability of sarcopenia patients to clear free radicals is reduced, and the excessive free radicals not being timely cleared post‐surgery could exacerbate tissue damage. Sarcopenia may also make patients more prone to falls, further aggravating the condition of sarcopenia [3].

Frailty is a multi‐dimensional, multifactorial condition involving physical function, cognitive abilities and psychosocial independence. A study emphasized the significance of the Frailty Index in predicting all‐cause and specific cause mortality rates among Chinese adults [4]. This research utilized a Frailty Index tailored for the Chinese population to assess patients' frailty status and found that frailty is also an independent risk factor for postoperative complications. This discovery aligns with the findings of Shaw, who identified a correlation between frailty and adverse outcomes in patients following cancer surgery [5]. The conclusion that sarcopenia and frailty act as independent risk factors is supported by the study that underscored the importance of jointly assessing these two conditions in predicting mortality [6].

Sarcopenia and frailty share some common characteristics, such as a high prevalence in the elderly population, a strong association with adverse health outcomes and potential reversibility. Although there are overlaps between the two, they are not equivalent concepts. Sarcopenia is considered a biological basis of frailty; however, not all individuals with sarcopenia will develop frailty. Studies show that 34.6% of sarcopenia patients are not frail, and 54.9% of frail patients do not have sarcopenia. Sarcopenia is primarily assessed through muscle mass, muscle strength and physical function; meanwhile, frailty encompasses a broader range of dimensions, including physical, psychological, social and cognitive aspects. There is a significant overlap between sarcopenia and frailty when assessing physical frailty. Sarcopenia and frailty are two distinct conditions, with sarcopenia not simply being a component of frailty.

The conclusion of the paper not only highlighted the negative impact of sarcopenia and frailty on postoperative recovery but also emphasized the importance of other factors such as gender, BMI, preoperative albumin levels and surgical stress scores. A study demonstrated that hypoalbuminemia was an independent predictor of various postoperative complications, echoing the findings in our study where albumin levels were identified as an independent risk factor [7]. The strength of our research lies in its grounding in the actual postoperative situations of elderly patients, taking into account physiological changes brought about by aging, such as endocrine function alterations, increased oxidative stress, inflammation, chronic diseases and malnutrition [8]. These changes make older adults more vulnerable to stress, leading to a reduction in physiological reserves across multiple organs and a diminished capacity to maintain homeostasis. Moreover, the study covers a variety of surgical types and their different degrees of surgical stress to comprehensively assess the impact of sarcopenia and frailty on elderly patients.

However, we must question the validity of this study as it appears to overlook the necessary stratification analysis for sarcopenia and frailty, which could lead to the absence of targeted interventions, confusion in study results, misestimation of risks, omission of crucial information and limitations in the evaluation of treatment effects. Stratification analysis can help accurately identify which patients most require specific types of interventions and reveal specific patterns or trends at different severity levels, which is very important for understanding disease progression and predicting prognosis. Sarcopenia can be categorized into non‐sarcopenia, sarcopenia and severe sarcopenia, while frailty can be divided into robust, pre‐frail and frail [4, 9]. By stratifying sarcopenia and frailty, we can more accurately assess the risks faced by patients at different levels and provide a basis for developing personalized treatment plans. Furthermore, stratification analysis helps to reveal the different pathophysiological mechanisms that may exist at different levels of sarcopenia and frailty and how these mechanisms impact postoperative recovery. This is crucial for guiding the selection of intervention measures, evaluating the effects of interventions and understanding the impact of different levels of sarcopenia and frailty on the long‐term health of patients.

Furthermore, the article mentioned that frailty assessment tools based on Western standards may not be suitable for the Chinese population. Although this study utilized a Frailty Index tailored for the Chinese population, the validity and reliability of this tool require further verification. While the study employed the Frailty Index to assess frailty status, this tool is limited to physical assessment and does not cover aspects of frailty related to psychological, social and cognitive dimensions. This could lead to a limited understanding of frailty. The study, although focusing on objective indicators such as the incidence of complications and hospital stay duration, lacks an assessment of patients' subjective experiences (e.g., pain levels, quality of life).

The novelty of this study lies in its combination of sarcopenia and frailty to explore their joint impact on postoperative recovery. This approach not only enriches our understanding of these two conditions in clinical practice but also provides a scientific basis for developing interventions targeting these conditions. However, there are limitations, such as the lack of stratification analysis, which could lead to the absence of targeted interventions, misestimation of risks and limitations in the evaluation of treatment effects. Furthermore, frailty assessment tools based on Western standards may not fully capture the multifaceted nature of frailty, especially lacking in the assessment of psychological, social and cognitive aspects. Future studies should incorporate Patient‐Reported Outcomes to better understand the overall patient experience.

## Conflicts of Interest

The authors declare no conflicts of interest.
